# Heritability of cortisol response to confinement stress in European sea bass *dicentrarchus labrax*

**DOI:** 10.1186/1297-9686-44-15

**Published:** 2012-06-19

**Authors:** Filip AM Volckaert, Bart Hellemans, Costas Batargias, Bruno Louro, Cécile Massault, Jeroen KJ Van Houdt, Chris Haley, Dirk-Jan de Koning, Adelino VM Canario

**Affiliations:** 1 Laboratory of Biodiversity and Evolutionary Genomics, KU Leuven, Ch. Deberiotstraat 32, B-3000, Leuven, Belgium; 2 Laboratory of Applied Fish Genetics and Fish Breeding, Department of Aquaculture & Fisheries Management, Technological Educational Institute of Messolonghi, Nea Ktiria, 30200, Messolonghi, Greece; 3 Centre of Marine Sciences (CCMAR), University of Algarve, Gambelas, P-8005-139, Faro, Portugal; 4 Division of Genetics and Genomics, Roslin Institute and Royal (Dick) School of Veterinary Sciences, University of Edinburgh, Roslin, Midlothian, EH25 9PS, Edinburgh, UK; 5 Animal Breeding and Genetics Group, Wageningen University, Postbox 338, NL-6700AH, Wageningen, The Netherlands; 6 Current address: Department of Animal Breeding and Genetics, Swedish University of Agricultural Sciences, 750 07, Uppsala, Sweden; 7 Current address: Laboratory for Cytogenetics and Genome Research, KU Leuven, Herestraat 49, B-3000, Leuven, Belgium

## Abstract

**Background:**

In fish, the most studied production traits in terms of heritability are body weight or growth, stress or disease resistance, while heritability of cortisol levels, widely used as a measure of response to stress, is less studied. In this study, we have estimated heritabilities of two growth traits (body weight and length) and of cortisol response to confinement stress in the European sea bass.

**Findings:**

The F1 progeny analysed (n = 922) belonged to a small effective breeding population with contributions from an unbalanced family structure of just 10 males and 2 females. Heritability values ranged from 0.54 (±0.21) for body weight to 0.65 (±0.22) for standard body length and were low for cortisol response i.e. 0.08 (±0.06). Genetic correlations were positive (0.94) between standard body length and body weight and negative between cortisol and body weight and between cortisol and standard body length (−0.60 and −0.55, respectively).

**Conclusion:**

This study confirms that in European sea bass, heritability of growth-related traits is high and that selection on such traits has potential. However, heritability of cortisol response to stress is low in European sea bass and since it is known to vary greatly among species, further studies are necessary to understand the reasons for these differences.

## Findings

Farming of European sea bass (*Dicentrarchus labrax,* Moronidae, Teleostei), represents about 100 000 tons produced per year [1] and attracts extensive interest as a major fish species for establishing breeding programmes to improve production traits. In fish, the most studied production traits in terms of heritability are body weight or growth, stress or disease resistance [2-6].

In this work, our aim was to set up European sea bass families by assigning parentage and heritability for three traits i.e. cortisol response to stress, body weight and standard body length to the progeny derived from the batch of a single spawning day.

## Methods

The methodology used for producing, phenotyping and genotyping the F1 population has been described by Massault et al [7]. In summary, the broodstock consisted of 34 females, 22 males and one individual of undetermined sex originating from wild and caged fish. From this broodstock, 2000 offspring were raised for 254 days under standard farm conditions and then distributed into four tanks of 45 m^3^, each with a net covering the inner surface. After this period of acclimatization, a confinement stress was applied, which consisted in slowly pulling the inner net of each tank so that the fish were confined in a volume of approximately 0.2 m^3^. After 4 h of confinement, the net was lifted and emptied into a tank of icy water, a process which stuns the fish within 3 min. Each group of 500 fish was numbered serially, bled, weighed and digitally photographed within 140 min after stunning, either in the morning (11 am) or afternoon (3 pm). Blood plasma was stored at −20°C for cortisol analysis and red blood cells stored in absolute ethanol for genetic analysis. Cortisol was measured by radioimmunoassay and microsatellite genotyping was carried out with three multiplex PCR (polymerase chain reaction) [See Additional file  1. All pedigree genotypes from the 11 larger families (n = 922) were checked for Mendelian errors before estimating heritabilities and correlations. Parentage assignment was implemented using three software packages [i.e., CERVUS v.3.0; [8], PAPA v.2.0; [9], VITASSIGN v.1.0; [10]] in order to constitute families with the highest possible certainty. The genotyping error rate was set to 1%. The assignment was tested for power and performance and locus-specific polymorphism information content (PIC) values were calculated.

Heritabilities and phenotypic correlations were calculated using phenotypic data collected on 930 animals. Eight animals were removed because of missing phenotypes. Thus, the dataset used to estimate heritability values comprised 922 animals, with missing values in some variables (see Table 1). Heritabilities were estimated using ASReml fitting an animal model. Several fixed effects were tested (sample set, day, tube number and assay number in cortisol assays) to check if they influenced the trait in question. With the exception of cortisol, the model was

(1) TRAIT=μ+A+E.

**Table 1:** Phenotypic traits for which genetic parameters were estimated in European sea bass

**Trait (unit)**	**Abbreviation**	**Mean**	**Standard deviation**	**Number of individuals**	**Coefficient of variation (%)**
Body weight (g)	BW	41.6	14.31	914	34.4
Standard length (cm)	SL	13.4	1.60	876	11.9
Cortisol (ng.ml^-1^)	CORT	318.5	141.36	713	44.4

Traits, abbreviation and measurements of mean, standard deviation and coefficient of variation.

*TRAIT* represents the phenotypic trait, *μ* the trait mean, *A* the additive genetic effect and *E* the environmental effect. Only sample set was found to have an effect on cortisol and therefore *sample set* was added as fixed effect to the model.

Phenotypic correlations were calculated with the software genstat v.10.

### Parentage assignment and contributions

The 1151 progeny and 56 parents were genotyped at 29 microsatellite loci. The number of alleles per locus varied between two and 10 and PIC values varied between 0.124 and 0.767 [see Additional file 2]. A power analysis was conducted with the rarefaction method and showed that 10 loci were sufficient for a reliable assignment (details not shown). A core group of five families contributed 71.5% to the progeny, six families made a measurable contribution and 80 families only a very small contribution. This is a highly skewed family representation of at least 748 dam x sire combinations with a low effective breeding size, which might affect the estimates through the unwanted genetic drift (or limited Mendelian sampling) caused by the skewed representation. For the heritability and correlation analyses, 922 offspring were used from which two females (5.9%) and 10 males (45.5%) contributed the most [see Additional file 3]. Our study shows that within a single breeding day, the majority of the progeny can be produced with the contribution of just two females and 10 males, which amounts to an effective population size (Ne) of 6.7 while there were 43 participating breeders and a total number of initial individuals of 57 (Ne/N = 0.12). Thus, artificial insemination provides the best guarantee to set up experimental crosses since during natural spawning the number of families with significant contribution can be small.

### Phenotypes

**Figure 1:**
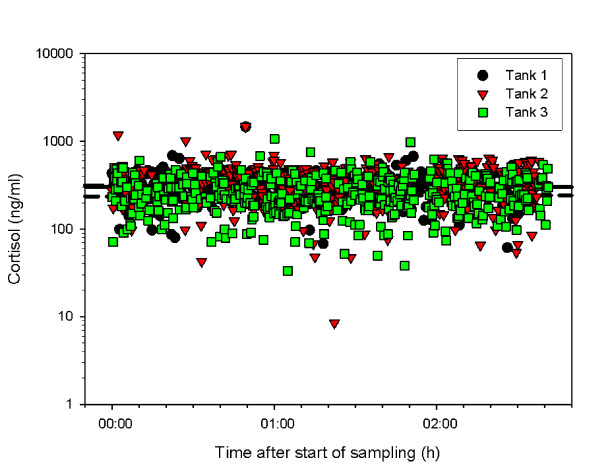
**Scatter plot of distribution of sea bass plasma cortisol in relation to sampling time.** Tanks 1, 2 and 3 represent groups of fish analyzed from different tanks (n = 1687); please note that the Y-axis is on a log scale; the slopes of the three curves range between −0.15 and 0.11 (7.64 × 10^-4^ < r^2^ < 2.11 × 10^-3^).

Basic descriptive statistics for the phenotypes are shown in Table 1. Mean cortisol levels were constant over the time of blood collection as indicated by the horizontal regression lines in Figure 1 (range of coefficients of linear regression per tank −0.153 to 0.109; r^2^ = 2.10^-3^).

### Heritabilities and correlations

Heritability values for growth traits ranged from 0.54 ± 0.21 for BW to 0.65 ± 0.22 for SL (Table 2). Such heritability estimates support the large proportion of phenotypic variation explained by the QTL detected in Massault et al. [7] and the reasons are discussed therein. For response to stress, the heritability of CORT was 0.08 ± 0.06. However, as noted above, the family structure is clearly suboptimal to estimate heritabilities as evidenced by the high standard errors of the estimates [11]. As expected, the phenotypic correlation between BW and SL was high (0.94), whereas CORT was not phenotypically correlated to either growth trait. The genetic correlation between SL and BW was high (0.94) and that between CORT and SL or BW was negative (−0.55 and −0.60, respectively). These estimates confirm data from previous studies in European seabass [12], rainbow trout [13] and Nile tilapia [14]. The value for body weight heritability obtained in our study also agrees with estimates previously reported in European sea bass, which range from medium heritabilities (0.2 in [12], 0.38-0.44 in [15] and 0.39 in [16,17]) to high heritabilities when taking into account different environments (0.31-0.60 in [18]). Generally, body length and body weight have moderate to high heritability values in teleost fishes: 0.6 in Coho salmon, *Oncorhynchus kisutch*[19], 0.12-0.47 in brown trout, *Salmo trutta*[20], 0.09-0.44 in carp, *Cyprinus carpio*[21,22], 0.38-0.79 in Nile tilapia, *Oreochromis niloticus*[23,24], 0.64 (± 0.12) in cod, *Gadus morhua*[25], 0.38 ± 0.07 in gilthead seabream, *Sparus aurata*[26].

**Table 2:** Heritabilities (bold), genetic correlations (upper triangle) and phenotypic correlations (lower triangle) and standard deviations (in brackets) for different traits in European bass (n 922)

BW		CORT	SL
BW	**0.54 (±0.21)**	−0.60 (±0.44)	0.94 (±0.07)
CORT	−0.04 (±0.05)	**0.08 (±0.06)**	−0.55 (±0.44)
SL	0.94 (±0.04)	−0.05 (±0.06)	**0.65 (±0.22)**

*BW* Body weight, *CORT* Cortisol response, *SL* Standard length.

To our knowledge, studies on the heritability of cortisol response to stress in fish have been limited to salmonids [27-29] and cyprinids [21], which limits generalizations. In addition, lines with high and low cortisol response to stress have been selected in rainbow trout [29]. However, the heritability of cortisol response to stress appears to be variable even among related species: 0.27-0.50 in rainbow trout, *Oncorhynchus mykiss*[27,29,30] and 0.60 in carp [21], but only 0.05 in Atlantic salmon *Salmo salar*[27,31]. These discrepancies can be partly explained by the differences in species and methodologies used to determine the cortisol response. It should be noted that the time-dependence of cortisol response to stress is a key element and a potential source of error. However, the methodology used in our study seems reliable since no apparent drift in cortisol levels with time was observed after applying the confinement stress (Figure 1). A comparative study on stunning methods used in different fish farms for European sea bass reported mean levels of cortisol response similar to that obtained here, corresponding to a 5-fold increase in cortisol compared to resting values when using ice [32]. In a pilot study on gilthead seabream using the same methodology, a significant change in cortisol levels of a control group not subjected to stress was observed, but there was no significant additional effect of ice-water on a group subjected to confinement stress (A. Canario, unpublished observations). Thus, the methodology adopted here for European sea bass is appropriate and even 2 h after stunning, the levels of cortisol obtained are due to the response to confinement stress and should reflect individual variation. In conclusion, in European sea bass, the growth traits measured have a moderate to high heritability but the cortisol level, as an indicator of response to stress, has a low heritability. Whether this low heritability derives from an artefact or an unbalanced family structure or whether it has a true biological base needs further clarification.

## Competing interests

The authors declare no competing interests.

## Authors’ contributions

FAMV planned the genotyping and wrote the manuscript, BH performed the genotyping, CB participated in the experimental planning, organized and carried out the experiment, participated in the sampling and wrote the paper, BL participated in the sampling and genotyping, CM performed the heritability and correlation analyses, JKJVH participated in the experimental planning and genetic analysis, CH participated in the experimental planning and genetic analysis, D-JK participated in the experimental planning, genetic analysis and wrote the manuscript, AVMC participated in the experimental planning, participated in the sampling, performed the cortisol analyses and wrote the manuscript. All authors read and approved the final manuscript.

## Supplementary Material

Additional file 1 **Multiplex assignment to linkage group.** Loci included in each multiplex set and assigned to *D. labrax* linkage groups, primer concentrations and comments on the 98 microsatellite markers used to scan the genome of European sea bass. Click here for file

Additional file 2 **Parentage assignment statistics.** Locus-specific PIC (Polymorphic Information Content) values, test of Hardy-Weinberg equilibrium (**: P < 0.05), null alleles, cumulative parentage assignment (in percent) with one and no parent known at high (95%) and low (80%) stringency. Click here for file

Additional file 3 **Consensus pedigree.**resspecies ID code of male and female parent, and number of offspring of the 11 largest families (FS01 to FS11) of the experimental population of European sea bass. The pedigree is based on assignments obtained with the software packages cervus, papa, and vitassign, and submission to the resspecies database (n = 922). Click here for file
