# Supplementary material for: Heritability of cortisol response to confinement stress in European sea bass dicentrarchus labrax
Source: Genet Sel Evol. 2012 Jun 19;44(1):15. doi: 10.1186/1297-9686-44-15 (PMC3378454; doi:10.1186/1297-9686-44-15)
Supplement: Additional file 1 — Multiplex assignment to linkage group. Loci included in each multiplex set and assigned to D. labrax linkage groups, primer concentrations and comments on the 98 microsatellite markers used to scan the genome of European sea bass. [file 1297-9686-44-15-S1.docx]

**Additional file 1 – Multiplex assignment to linkage group**

Loci included in each multiplex set assigned to *D. labrax* linkage groups, primer concentration and remarks of the 98 microsatellite markers used to scan the genome of European sea bass.

| Multiplex set | Linkage Group | Primer (µM) | Remark |
| --- | --- | --- | --- |
| **Multi01** |  |  |  |
| DLA0275e | 6 | 0.025 | monomorphic |
| DLA0051 | 1 | 0.05 |  |
| DLA0049 | 7 | 0.05 |  |
| DLA0021 | 1 | 0.05 |  |
| DLA0162 | 1 | 0.05 |  |
| DLA0164 | 1 | 0.1 |  |
| DLA0122 | 1 | 0.1 |  |
| DLA0038 | 1 | 0.2 |  |
| DLA0016 | 1 | 0.4 |  |
| DLA0233e | 8 | 0.4 |  |
| **Multi02** |  |  |  |
| DLA0237PY | 1 | 0.025 |  |
| DLA0167 | 1 | 0.025 |  |
| DLA0149 | 5 | 0.025 |  |
| DLA0009 | 7 | 0.025 |  |
| DLA0166 | 4 | 0.025 |  |
| DLA0273e | 10 | 0.025 |  |
| DLA0200 | 2 | 0.05 |  |
| DLA0251e | 1 | 0.05 |  |
| DLA0104 | 2 | 0.05 |  |
| DLA0118 | 2 | 0.05 |  |
| DLA0036 | 2 | 0.1 |  |
| DLA0106 | 2 | 0.4 |  |
| **Multi03** |  |  |  |
| DLA0254PXN1 | 16 | 0.025 | monomorphic |
| DLA0131 | 2 | 0.05 |  |
| DLA0032 | 5 | 0.05 |  |
| DLA0039 | 2 | 0.05 |  |
| DLA0040 | 14 | 0.05 |  |
| DLA0133 | 3 | 0.05 |  |
| DLA0267e | 11 | 0.1 |  |
| DLA0272e | 6 | 0.1 |  |
| DLA0026 | 3 | 0.2 |  |
