# Supplementary material for: Heritability of cortisol response to confinement stress in European sea bass dicentrarchus labrax
Source: Genet Sel Evol. 2012 Jun 19;44(1):15. doi: 10.1186/1297-9686-44-15 (PMC3378454; doi:10.1186/1297-9686-44-15)
Supplement: Additional file 2 — Parentage assignment statistics. Locus-specific PIC (Polymorphic Information Content) values, test of Hardy-Weinberg equilibrium (**: P < 0.05), null alleles, cumulative parentage assignment (in percent) with one and no parent known at high (95%) and low (80%) stringency. [file 1297-9686-44-15-S2.docx]

**Additional file 2 – Parentage assignment statistics**

Locus-specific Polymorphic Information Content (PIC) values, test of Hardy-Weinberg equilibrium (**: P < 0.05), null alleles, cumulative parentage assignment (in percent) with one and none parent known at high (95%) and low (80%) stringency.

| Locus number | Locus | PIC | HW | Null | Stringency with one parent known | | Stringency with no parent known | |
| --- | --- | --- | --- | --- | --- | --- | --- | --- |
|  |  |  |  |  | 95% | 80% | 95% | 80% |
| 1 | DLA0009 | 0.767 | ** | +0.0281 | 0 | 0 | 0 | 0 |
| 2 | DLA0038 | 0.763 | ** | +0.2088 | 0 | 5 | 0 | 0 |
| 3 | DLA0167 | 0.740 | ** | -0.0390 | 8 | 25 | 0 | 0 |
| 4 | DLA0039 | 0.725 | ** | -0.0358 | 26 | 53 | 1 | 5 |
| 5 | DLA0032 | 0.715 | ** | -0.0442 | 46 | 86 | 1 | 20 |
| 6 | DLA0122 | 0.691 | ** | -0.0466 | 66 | 100 | 10 | 40 |
| 7 | DLA0131 | 0.686 | ** | -0.0123 | 79 | 100 | 16 | 58 |
| 8 | DLA0016 | 0.677 | ** | -0.0434 | 90 | 100 | 31 | 77 |
| 9 | DLA0164 | 0.645 | ** | +0.1419 | 93 | 100 | 46 | 88 |
| 10 | DLA0118 | 0.641 | ** | -0.0151 | 96 | 100 | 56 | 98 |
| 11 | DLA0040 | 0.630 | ** | -0.0162 | 99 | 100 | 67 | 100 |
| 12 | DLA0233e | 0.626 | ** | +0.0047 | 99 | 100 | 73 | 100 |
| 13 | DLA0049 | 0.605 | ** | -0.0093 | 100 | 100 | 76 | 100 |
| 14 | DLA0021 | 0.588 | ** | -0.0254 | 99 | 99 | 85 | 100 |
| 15 | DLA0149 | 0.580 | ** | +0.0061 | 99 | 99 | 88 | 100 |
| 16 | DLA0036 | 0.562 | ** | +0.1446 | 99 | 99 | 90 | 100 |
| 17 | DLA0051 | 0.526 | ** | -0.0244 | 99 | 99 | 93 | 100 |
| 18 | DLA0162 | 0.513 | ** | -0.0146 | 99 | 99 | 93 | 100 |
| 19 | DLA0133 | 0.484 | ** | +0.0111 | 99 | 99 | 95 | 100 |
| 20 | DLA0104 | 0.414 | ** | -0.0401 | 99 | 99 | 96 | 100 |
| 21 | DLA0237PY | 0.375 | NS | +0.0151 | 98 | 98 | 96 | 100 |
| 22 | DLA0026 | 0.375 | ** | +0.0484 | 99 | 99 | 97 | 100 |
| 23 | DLA0166 | 0.371 | ** | -0.1016 | 98 | 98 | 97 | 100 |
| 24 | DLA0106 | 0.351 | NS | -0.0320 | 98 | 98 | 98 | 100 |
| 25 | DLA0251e | 0.330 | ** | +0.9059 | 98 | 98 | 98 | 100 |
| 26 | DLA0267e | 0.324 | ** | -0.0892 | 98 | 98 | 98 | 100 |
| 27 | DLA0273e | 0.312 | ** | -0.0577 | 98 | 98 | 98 | 100 |
| 28 | DLA0200 | 0.277 | NS | -0.0247 | 98 | 98 | 98 | 100 |
| 29 | DLA0272e | 0.124 | NS | -0.0311 | 98 | 98 | 98 | 100 |
