# Supplementary material for: Heritability of cortisol response to confinement stress in European sea bass dicentrarchus labrax
Source: Genet Sel Evol. 2012 Jun 19;44(1):15. doi: 10.1186/1297-9686-44-15 (PMC3378454; doi:10.1186/1297-9686-44-15)
Supplement: Additional file 3 — Consensus pedigree.resspecies ID code of male and female parent, and number of offspring of the 11 largest families (FS01 to FS11) of the experimental population of European sea bass. The pedigree is based on assignments obtained with the software packages cervus, papa, and vitassign, and submission to the resspecies database (n = 922). [file 1297-9686-44-15-S3.docx]

**Additional file 3 - Consensus pedigree**

ResSpecies ID code of male and female parent, and number of offspring of 11 largest families (FS01 to FS11) of the experimental population of European sea bass. The pedigree is based on assignment with the software packages Cervus, Papa, and Vitassign, and submission to the ResSpecies database (n = 922).

|  | **FS01** | **FS02** | **FS03** | **FS04** | **FS05** | **FS06** | **FS07** | **FS08** | **FS09** | **FS10** | **FS11** |
| --- | --- | --- | --- | --- | --- | --- | --- | --- | --- | --- | --- |
| **Sire ID** | 72 | 73 | 74 | 77 | 78 | 1080 | 74 | 1082 | 1085 | 1086 | 1088 |
| **Dam ID** | 76 | 75 | 76 | 76 | 75 | 75 | 75 | 76 | 75 | 75 | 75 |
| **# offspring** | 92 | 99 | 338 | 195 | 92 | 8 | 13 | 14 | 17 | 15 | 39 |
